# Influence of Pasteurization and Storage on Dynamic In Vitro Gastric Digestion of Milk Proteins: Quantitative Insights Based on Peptidomics

**DOI:** 10.3390/foods9080998

**Published:** 2020-07-25

**Authors:** Xing Li, Yuxiang Gu, Shudong He, Olayemi Eyituoyo Dudu, Qiming Li, Haiyan Liu, Ying Ma

**Affiliations:** 1 School of Chemistry and Chemical Engineering, Harbin Institute of Technology, 92 West Dazhi Street, Harbin 150001, China; leestar1104@163.com (X.L.); Yuxiang.Gu@outlook.com (Y.G.); duduolayemi@live.com (O.E.D.); liuhy1@newhope.cn (H.L.); 2 School of Food and Biological Engineering, Engineering Research Center of Bio-Process of Ministry of Education, Hefei University of Technology, Hefei 230009, China; shudong.he@hfut.edu.cn; 3 New Hope Dairy Co, Ltd., Chengdu 610063, China; qm258@126.com; 4 Dairy Nutrition and Function, Key Laboratory of Sichuan Province, Chengdu 610000, China

**Keywords:** milk protein, dynamic in vitro digestion, peptidomics, pasteurization milk, storage

## Abstract

It is important to evaluate the nutritional quality of milk during the shelf-life, especially during home storage, from a consumer viewpoint. In this study, we investigated the impact of pasteurization (85 °C/15 s) and subsequent storage (at 4 °C for 7 days) on the coagulation behavior of milk and protein digestibility in a dynamic in vitro gastric digestion test. A high level of hydration in curd formed in pasteurized milk upon 7-day cold storage compared to raw and pasteurized milk, indicating fast pepsin diffusion in the interior of curds, increasing the hydrolysis rate. The digesta collected at various time points throughout the gastric digestion were studied using *o*-phthaldialdehyde (OPA), sodium dodecyl sulfate polyacrylamide gel electrophoresis (SDS-PAGE), liquid chromatography tandem mass spectrometry (LC-MS/MS), and amino acid analysis. These results showed that milk proteins were hydrolyzed quickly upon a long period of cold storage. Additionally, qualitative and quantitative results obtained using LC-MS/MS exhibited significant differences between samples, especially in pasteurized milk upon cold storage. Processing and storage played a decisive role in bioactive peptide generation. Such knowledge could provide insights into and directions for the storage of pasteurized milk for further clinical studies on protein bioavailability and the generation of bioactive peptides for desired health outcomes.

## 1. Introduction

Milk proteins, as an important aspect of dietary proteins for humans, have a good nutritional value: They have a high content of several essential amino acids and good digestibility [1]. Moreover, milk proteins have recently attracted an increasing amount of attention, since they contain bioactive peptides which can be released during gastrointestinal digestion [2]. Caseins, representing 80% of milk proteins, form a highly hydrated micellar assembly of several thousand individual casein molecules, known as casein micelles [3]. β-lactoglobulin (β-Lg), a major whey protein, has been extensively studied because of its thermal instability. Heat treatment above 60 °C leads to β-Lg denaturation, aggregation, and the association of denatured β-Lg with casein micelles through interactions with κ-casein [4]. These structural changes at the molecular level can lead to changes in the physicochemical properties of milk (viscosity, surface hydrophobicity, rheological property, etc.) [5,6,7]. Moreover, these changes might have both desirable and undesirable effects on the cleavage sites of enzymes, resulting in the modification of protein digestibility.

Milk proteins show complex digestive kinetics during gastric digestion. Casein micelles can precipitate and coagulate curds during gastric digestion. A reduction of the pepsin diffusivity inside curds compared to diffusion in solution can significantly decrease the rate of gastric emptying [6]. Whey proteins, which are soluble in serum, can remain mostly intact during gastric digestion and quickly enter the duodenum. However, heat treatment > 80 °C induces the unfolding of β-Lg, which exposes the peptic cleavage sites and thus increases their susceptibility to hydrolysis [8]. The digestibility of casein is also altered upon heat treatment [9,10]. The impact of heat treatment on changes in the cleavage sites of milk proteins using mass spectrometry have attracted extensive attention [10,11,12]. The results have shown that the effect of heat treatment can notably change the peptides released during gastric digestion. Regrettably, no quantitative data have been obtained because of limited mass spectrometry technology, so there is no clear guidance for evaluating the differences in cleavage sites. Ye, Cui (9) found that the curds formed in heated milk during gastric digestion can be hydrolyzed quickly compared to raw milk using sodium dodecyl sulfate polyacrylamide gel electrophoresis (SDS-PAGE) based on a dynamic gastric simulator.

As a major commercial dairy product, pasteurized milk is consumed globally in large quantities. Pasteurized milk is not sterile. Therefore, it needs to be stored at 4–6 °C and has a shelf-life of approximately 7 days from a microbiological safety point of view [13]. Some association and dissociation of milk proteins occur in pasteurized milk during storage, despite the short shelf-life and low storage temperature [14,15]. Modification of the surface hydrophobicity of proteins and a marked increase of the casein micelle size were observed in pasteurized milk upon cold storage [15]. These results showed that protein interactions still occurred in a prolonged storage time, which might influence the milk protein bioavailability.

Little is known about the changes in milk protein digestibility that occur in pasteurized milk during storage. From a nutritional point of view, the effects of the storage time on milk protein digestibility could influence the nutritional quality of the product. In this study, we investigated the impact of storage on the hydrolysis kinetics of proteins in pasteurized milk, with a particular focus on the evolution of the peptidome generated during gastric digestion under these conditions. The recent advent of highly sensitive mass spectrometry (Q Exactive mass spectrometry (ThermoFisher Scientific, San Jose, CA, USA) coupled with Maxquant software version 1.5.0.0) can provide site-specific identification and semi-quantification. The identification of these differences will provide a better understanding of the nutritional quality and deliver better benefits of liquid milk products.

## 2. Materials and Methods

### 2.1. Materials

Fresh raw milk (RM) and commercial skimmed pasteurized milk were obtained from a local dairy company (Harbin, Heilongjiang, China). Skimmed pasteurized milk was subjected to an indirect injection at 85 °C for 15 s in a commercial-scale pasteurized plant (Xuhui Co., Ltd., Shanghai, China). The defatted process was conducted using a milk defatting centrifugal cream separator (Xuhui Co., Ltd., Shanghai, China) prior to pasteurization. The batch size was 10 tons and the volumetric flow rate was 5 tons/h. Pasteurized milk was stored at 4 °C for up to 7 days. Samples were taken for analysis at day 0, 3, and 7 (PM0d, PM3d, and PM7d, respectively).

Pepsin from porcine gastric mucosa (P7000, 825 U/mg) was purchased from Sigma-Aldrich Corp. (St. Louis, MO, USA). Coomassie Brilliant Blue R-250 was obtained from Bio-Rad Laboratories, Inc. (Hercules, CA, USA). Water was purified by treatment with a Millipore purification system (>18.3 MΩ·cm, Millipore Corp., Bedford, MA, USA) and used for all experiments. All of the other chemicals obtained from Solarbio Co., Ltd. (Beijing, China) were of analytical grade, unless otherwise specified.

### 2.2. Dynamic In Vitro Gastric Digestion

**Figure 1:**
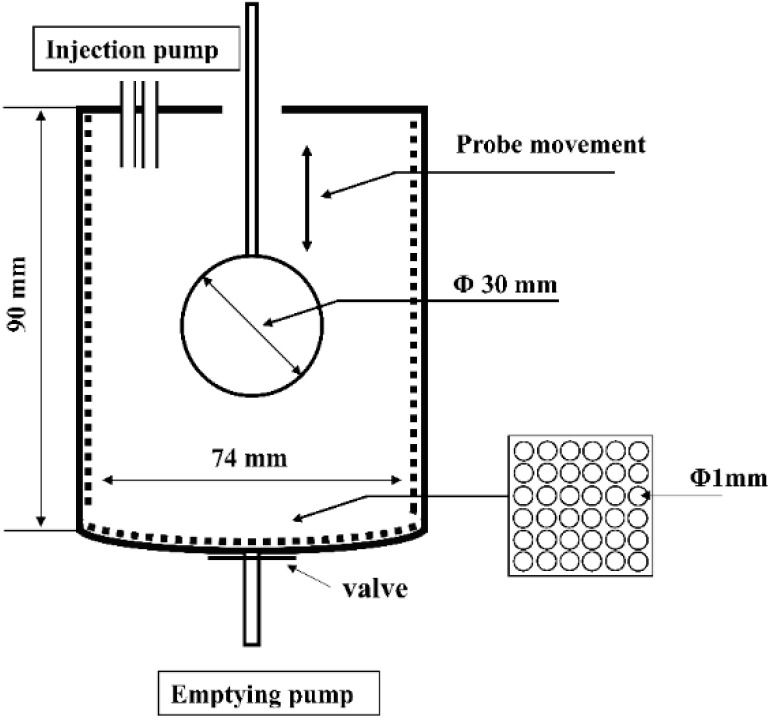
A schematic illustration of the dynamic in vitro gastric digestion model.

The dynamic gastric digestion model used for in vitro gastric digestion was developed according to the human gastric simulator (HGS), with some modification [16]. Simulated gastric fluid (SGF) stock electrolyte solution was adjusted to the recommended levels, as described in the standardized consensus [17]. The dynamic gastric digestion model (Figure 1) mainly consisted of a digestion beaker, a temperature-controlled incubator (HZS-HA, HDL Apparatus Institute, Changzhou, China), a spherical Teflon probe of a 30 mm diameter attached to the stepper motor via a thin Teflon rod of a 5 mm diameter, two injection pumps (F01A STP, Kamoer pump Co., Ltd., Shanghai, China), and an emptying pump (NKCP, Kamoer pump Co., Ltd., Shanghai, China). The main digestion beaker had a diameter of 74 mm, a depth of 90 mm, and a volume of 350 mL. A simple mechanical movement was able to simulate the peristaltic contractions of the stomach wall according to a published study, with modification [18]. The probe could be moved up and down under the control of the stepper motor at 9 mm/s to simulate the actual stomach contraction frequency of 3 cycles per minute [19]. The maximum antral force in this model was calculated using the mathematical modeling software COMSOL Multiphysics (COMSOL, Inc., Version 5.5, Stockholm, Sweden) and the maximum antral force was 23,000 N/m^2^, which was in a reasonable range of that presented in a human stomach ranging from 5134 to 67,292 N/m^2^ [16]. A thin polyester mesh bag (pore size 1 mm) was placed inside the digestion beaker to simulate a sieving effect of the pylorus, which only allowed particles with a size < 1 mm to pass through to the duodenum [20]. The temperature of the beaker was maintained at 37 °C using the temperature-controlled incubator.

The pH of the SGF was adjusted to 1.5 using 1 M HCl. Another simulated digestive fluid containing 4.8 g/L of pepsin and 0.15 mM of CaCl_2_ was diluted to the correct electrolyte concentration with water. The dosage of the SGF and pepsin solution was 80 and 20 mL, respectively, as described in our previous study [21]. They were then added at a rate of 2.5 mL/min (controlled by a rate of 2.0 mL/min of the SGF and a rate of 0.5 mL/min of the pepsin solution addition by using two injection pumps, respectively). All of the solutions were warmed at 37 °C. The milk sample (100 mL) was loaded into the digestion beaker and warmed at 37 °C for 2 min to mimic oral prewarming. The digesta began to be emptied out from 30 min and the gastric emptying rate was 3.0 mL/min. The gastric digestion lasted 2 h. Samples of the digesta were collected at 2, 5, 10, 20, 30, 60, 90, and 120 min of digestion for an analysis of breakdown products. Samples of the digesta were adjusted to 6.8 using dilute NaOH to terminate the enzymatic reaction. Samples at each time point were prepared separately, and all experiments were conducted in triplicate.

### 2.3. pH Measurement

The initial pH was defined as the pH of the milk. The pH at different digestion time points was defined as the pH of the emptied digesta according to the previous method [20].

### 2.4. Weight of Curds

The curds obtained from the raw milk and pasteurized milk after digestion at different time points were removed and weighted immediately [9]. The weights of dried curds were determined after being dried at 105 °C for 12 h in a vacuum oven [9,20].

### 2.5. Degree of Hydrolysis

The extent of protein hydrolysis was determined using the standardized *o*-phthaldialdehyde (OPA) spectrophotometric assay [22]. Briefly, 0.2 mL of sample was added to 1.5 mL of OPA reagent, mixed well, and incubated at room temperature for 2 min. The optimal density at 340 nm was measured using a UV-2100 spectrophotometer (Beifen-Ruili Analytical Instrument Co., Ltd., Beijing, China). The number of amino groups was determined from an L-leucine (0–2 mg/mL) standard curve and the proteolytic activity was calculated according to the equation described in a previous report [22]. Each assay was done in triplicate for all samples.

### 2.6. Sodium Dodecyl Sulfate Polyacrylamide Gel Electrophoresis (SDS-PAGE)

SDS-PAGE was performed as described by Ji, Li (23) using a 15% resolving gel and 4% stacking gel. All samples were centrifuged at 12,000× *g* for 10 min and supernatant fractions were mixed with SDS-PAGE buffer at a volume ratio of 1:1. The gel electrophoresis was run at 125 V for 1.5 h. The gels were imaged and quantified using the Molecular Imager Gel Doc XR system (Bio-Rad Laboratories, Hercules, CA, USA) and Quantity One 1-D analysis software (Bio-Rad Laboratories, Hercules, CA, USA).

### 2.7. Liquid Chromatography Tandem Mass Spectrometry (LC-MS/MS)

The digesta were filtered through 3000 molecular weight cut-off centrifugal filters (Amicon^®^ Ultra-4, Merck Millipore, Billerica, MA, USA) and equalized by adding an appropriate volume of water prior to running LC-MS. The Thermo Scientific Q Exactive mass spectrometer equipped with an UHPLC system (Easy-nLC^TM^ 1000, Thermo Fisher Scientific, San Jose, CA, USA) was applied for liquid chromatography tandem mass spectrometry (LC-MS/MS) analysis. A total of 10 µL of sample was loaded on a reverse phase trap column (Thermo Scientific Acclaim PepMap100, 200 mm × 100 μm, nanoViper C18, San Jose, CA, USA) connected to the C18-reversed analytical column (Thermo Scientific Easy Column, 3 μm, 100 mm × 75 μm, San Jose, CA, USA). The elution of peptides was carried out at a flow rate of 300 nL/min controlled by IntelliFlow technology (Thermo Fisher Scientific, San Jose, CA, USA), with solvent A (water containing 0.1% formic acid) and solvent B (84% acetonitrile containing 0.1% formic acid) as eluents. The gradient elution program was as follows: linear gradient from 0 to 35% B for 50 min, from 35 to 100% B for 5 min, and held at 100% B for 5 min.

The mass spectrometer was operated in positive ion mode. A spray voltage of 2.2 kV was used with a transfer capillary temperature of 200 °C. Mass Spectrometry (MS) data were acquired using a data-dependent top10 method dynamically choosing the most abundant precursor ions from the survey scan (300–1800 m/z) for high-energy collisional dissociation (HCD) fragmentation [23]. An automatic gain control (AGC) target was set to 3e6, and the maximum injection time was set to 10 ms. The dynamic exclusion duration was 40.0 s. Survey scans were acquired at a resolution of 70,000 at m/z 200, the resolution for HCD spectra was set to 17,500 at m/z 200, and the isolation width was 2 m/z. The normalized collision energy was 30 eV and the underfill ratio, which specifies the minimum percentage of the target value likely to be reached at the maximum fill time, was defined as 0.1%. The instrument was run with peptide recognition mode enabled.

### 2.8. Sequence Database Searching and Data Analysis

MS/MS spectra were searched using MaxQuant software version 1.3.0.5 (Max Planck Institute of Biochemistry, Martinsried, Germany) against the UniProt Bos Taurus database and the decoy database. For protein identification, the following parameters were set: Main search = 6 ppm, first search = 20 ppm, MS/MS tolerance = 20 ppm, and max missed cleavage = 2. The fixed modification was Carbamidomethyl (C) and the variable modifications were the oxidation (M), acetyl (protein N-term), protein false discovery rate (FDR) ≤ 0.01, and peptide FDR ≤ 0.01.

Peptigram, a web-based visualization tool for peptidomics data, was used to visualize the peptide distributions in different samples. It is freely available for academic use at http://bioware.ucd.ie/peptigram [24]. To investigate the differences in the bioactive peptides between different samples, the BIOPEP database (http://www.uwm.edu.pl/biochemia/index.php/en/biopep) was used to match peptide sequences.

### 2.9. Amino Acid Analysis

The levels of free amino acids released from samples were determined by ion chromatography, according to a previous report [25]. Briefly, 1 mL samples were mixed with 50 mg of sulfosalicylic acid and incubated for 1 h at 4 °C. The mixtures were centrifuged at 5000× *g* for 15 min at 4 °C and the supernatants were filtered through a 0.45 μm pore-size membrane (A-FIT Biosciences Ltd., Beijing, China). The filtrate was diluted two times with a 0.2 M lithium citrate buffer (pH 2.2). The amino acid analysis was then carried out by cation exchange chromatography on an automatic amino acid analyzer (Biochrom Ltd., Cambridge, Cambourne, UK).

### 2.10. Statistical Analysis

All samples were prepared in three independent milk aliquots and each was analyzed in triplicate. Data were analyzed by an analysis of variance (ANOVA) and the differences between means were analyzed by the Duncan test. The results were considered significant at *p* < 0.05.

## 3. Results and Discussion

### 3.1. Change in pH

**Figure 2:**
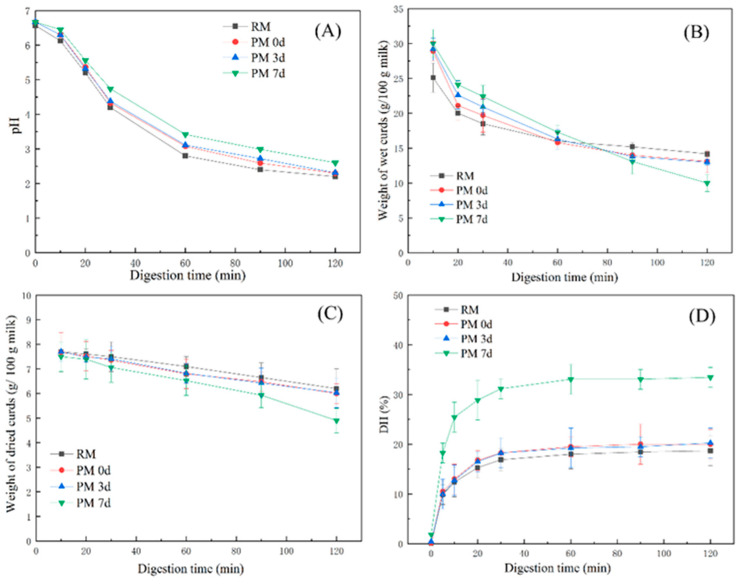
Changes in the pH (**A**), weight of wet curds (g/100 g milk) (**B**), weight of dried curds (g/100 g milk) (**C**), and degree of protein hydrolysis (**D**) from raw milk (RM), pasteurized milk (PM0d), pasteurized milk upon 3 days of storage (PM3d), and pasteurized milk upon 7 days of storage (PM7d) during in vitro gastric digestion. Error bars represent the standard deviation of three independent samples and each was measured in triplicate.

The pH of the samples gradually decreased along with an increase in the digestion time (Figure 2A). There was no significant difference in the pH profiles between pasteurized milk and raw milk, but a slower decrease in the pH was observed in pasteurized milk during storage. The differences in the pH profiles were ascribed to the different structures of the curds that formed in milk samples during digestion, which could alter the buffer capacity of milk [11,20]. A slow decrease in pH observed in pasteurized milk upon 7 days of storage indicated that a curd which could exert a greater buffer capacity was formed and slowed down the decrease in pH.

### 3.2. Coagulation Behavior of Milk

The weights of the curds decreased gradually with the digestion time up to 120 min (Figure 2B). During the early digestion period (at 10 min), the weights of the curds formed from pasteurized milk were greater than those in raw milk (*p* < 0.05). However, after 90 min of digestion, the curd weights from pasteurized milk, especially upon 7 days of cold storage, were lower than those from raw milk. Figure 2C shows that all milk samples had similar weight profiles of dried curds, except for the weight from pasteurized milk stored at 7 days, which was lower than other samples at 120 min of gastric digestion (*p* < 0.05).

The curds were visible at 10 min of digestion where the pH was above 6.0, which was consistent with previous reports on whole and skimmed milk [11,20]. This could be attributed to the destabilization of casein micelles caused by the proteolytic activity of pepsin. Casein micelles are stabilized by steric repulsion generated by the polyelectrolyte layer of κ-casein [5]. κ-casein has been shown to be hydrolyzed faster than other caseins by pepsin at pH 6.0 [26], which could induce sterical destabilization of the micelles and cause them to coagulate. At 10 min of gastric digestion, the weights of wet curds in pasteurized milk were higher than those in raw milk, while the weights of dry curd were similar between raw and pasteurized milk. This result indicated higher hydration of the curd formed in pasteurized milk, which could easily provide cleavage sites for pepsin and increase the digestion rate. The quick hydrolysis of curds observed in milk upon 7-day storage during gastric digestion could be due to the highest water-holding capacity of curds, which could be more susceptible to hydrolysis by pepsin.

### 3.3. The Degree of Protein Hydrolysis

Figure 2D shows the hydrolysis degree of milk proteins in samples. All of the samples were quickly hydrolyzed in the first 30 min of digestion and then remained constant after 60 min. At the end of the gastric digestion (120 min), a higher hydrolysis degree (33%) was observed from pasteurized milk upon 7 days of storage compared to other samples (19–20%). Milk proteins that existed in the interior of the curd could be hardly hydrolyzed by pepsin. The high level of hydration in the curd formed in pasteurized milk upon 7-day storage could provide faster pepsin diffusion in the curd, leading to a higher hydrolysis degree in the PM7d sample.

### 3.4. Hydrolysis of Protein in Digesta

**Figure 3:**
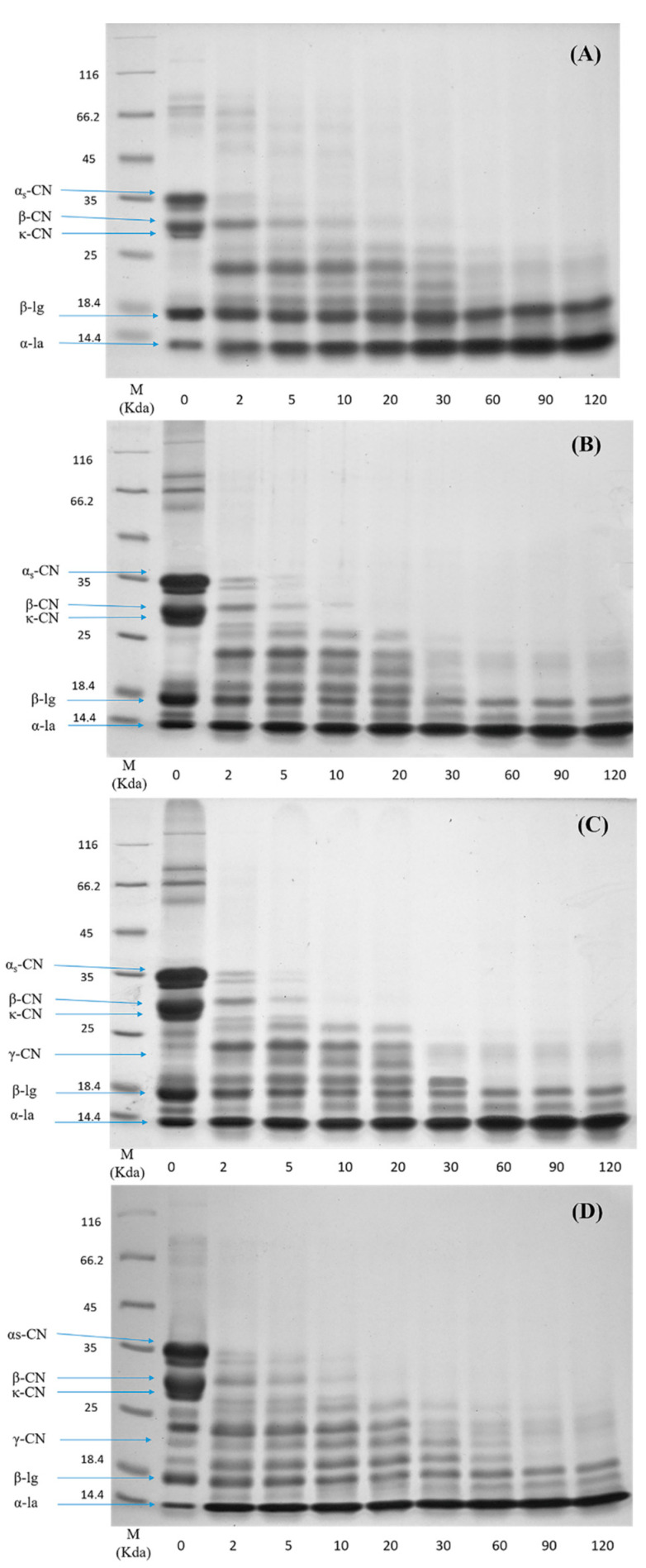
Sodium dodecyl sulfate polyacrylamide gel electrophoresis (SDS-PAGE) profiles of the in vitro gastric digestion of (**A**) raw milk, (**B**) pasteurized milk, (**C**) pasteurized milk upon 3 days of storage, and (**D**) pasteurized milk upon 7 days of storage. Lane M shows molecular weight markers. The number at the bottom of each lane represents the time of digestion (min).

To investigate the difference in the protein hydrolysis kinetics between the raw milk and pasteurized milk upon different storage times, the SDS-PAGE profiles of samples in the digesta at different time points of digestion are shown in Figure 3. In general, caseins were digested much faster than whey proteins under the in vitro gastric condition. In the case of raw milk (Figure 3A), caseins were rapidly hydrolyzed within the first 2 min of digestion and no obvious intact caseins were visible after 20 min. In comparison, the digestion of major whey protein β-Lg was much slower, as indicated by the small change in the intensity of the β-Lg band during the 120 min gastric digestion. No significant changes in the intensity of the casein bands in pasteurized milk (PM0d) were observed compared to raw milk (Figure 3B). The digestion of β-Lg was slightly increased in the pasteurized milk compared to raw milk during the initial 2 min and remained constant over 120 min of digestion. During the subsequent storage of pasteurized milk, the SDS-PAGE profiles of proteins were altered. The contents of caseins and β-Lg in stored pasteurized milk prior to digestion were decreased based on quantitative data, although there were minor changes in PM3d (at time 0 in Figure 3C,D). Moreover, several new intermediate molecular weight bands (19 to 24 kDa) were visible in stored pasteurized milk prior to digestion, following by gradual degradation over 30 min of digestion. The changes in the intensities of casein and β-Lg bands during digestion were not obvious in stored pasteurized milk compared with those in PM0d.

Caseins are susceptible to pepsin hydrolysis. Native β-Lg is more resistant to pepsin hydrolysis because of its structural stability at a low pH [27,28,29], as most of the hydrophobic amino acid residues, which are potential cleavage sites for pepsin, are buried inside the β-barrel and thus not readily accessible [9]. β-Lg is also more sensitive to heat-induced structural unfolding beginning at 80 °C [29]. The unfolding of β-Lg provided better excess for pepsin to hydrolyze, leading to a faster digestion rate of β-Lg [7]. The hydrolysis of β-Lg in pasteurized milk was limited, which was due to the low content of denatured β-Lg in pasteurized milk.

Relatively low contents of proteins in stored milk indicated that protein hydrolysis occurred in cold storage. This observation was consistent with our previous work, in which the protein content was decreased and non-protein nitrogen was increased during storage [15]. The digestion patterns of caseins and β-Lg in digesta were similar between PM0d and stored pasteurized milk. The mechanism behind this phenomenon was not clear, and it can probably be attributed to three major reasons. The first was that the cold storage promoted a small dissociation of colloidal caseins, especially β-CN [30]. The dissociated caseins that entered the serum could be easily hydrolyzed by pepsin. The second was that the curds in long-period stored milk were hydrolyzed quickly, which might also induce more small curd particles (<1 mm) peeled off into the digesta during the initial period of digestion. The particle size analysis using the Nano-ZS particle analyzer (Malvern Instruments Ltd., UK) showed a higher proportion of relatively large particles (>1000 nm) found in stored milk (result not known). These particles might result in an increase of the intact protein contents in digesta using SDS-PAGE analysis. The third was protein interactions and associated molecular modifications occurred and increased during storage, especially in pasteurized milk upon 7 days of storage [15,17], which could change the protein digestibility. For example, glycation and lactosylation could block lysine residues and thereby hinder the access of pepsin to the cleavage sites. Indeed, further work was needed to study the impact of cold storage on the digestibility of the individual milk protein. It was noted that γ-CN (at ~21 kDa) was observed in pasteurized milk during storage, indicating that β-CN was hydrolyzed in the prolonged storage time. γ-CN was more slowly hydrolyzed compared to other caseins and the band was observed after 30 min of digestion. Plasmin, a heat-stable proteinase that exists in pasteurized milk during storage, can cleave the plasmin-sensitive bonds in β-CN and lead to the formation of γ-CN [31]. The slow hydrolysis rate of γ-CN might be due to the fact that the structural changes in γ-CN originating from β-CN could hinder the access of pepsin to the cleavage sites. These results indicated that cold storage could change the milk protein digestibility.

### 3.5. Peptides Generated upon Cold Storage

**Figure 4:**
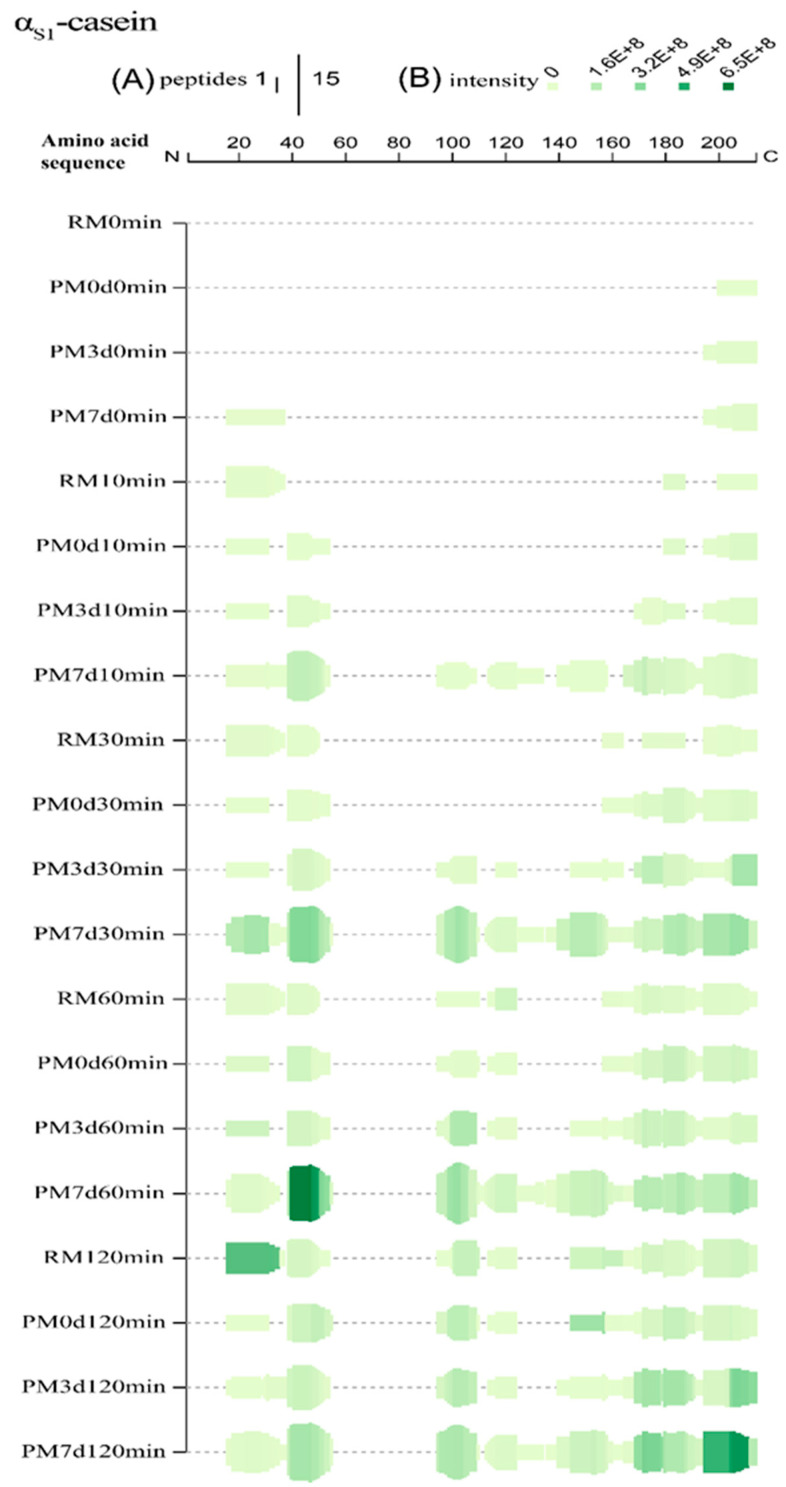
Peptide profiles originating from α_s1_-casein during the in vitro gastric digestion of raw milk (RM), pasteurized milk (PM0d), pasteurized milk upon 3 days of storage (PM3d), and pasteurized milk upon 7 days of storage (PM7d). (**A**) At each amino acid residue along the protein, the height of the green bars is proportional to the count of peptides overlapping this position; (**B**) the intensity of the color (green) is proportional to the sum of the peptide intensities overlapping this position.

**Figure 5:**
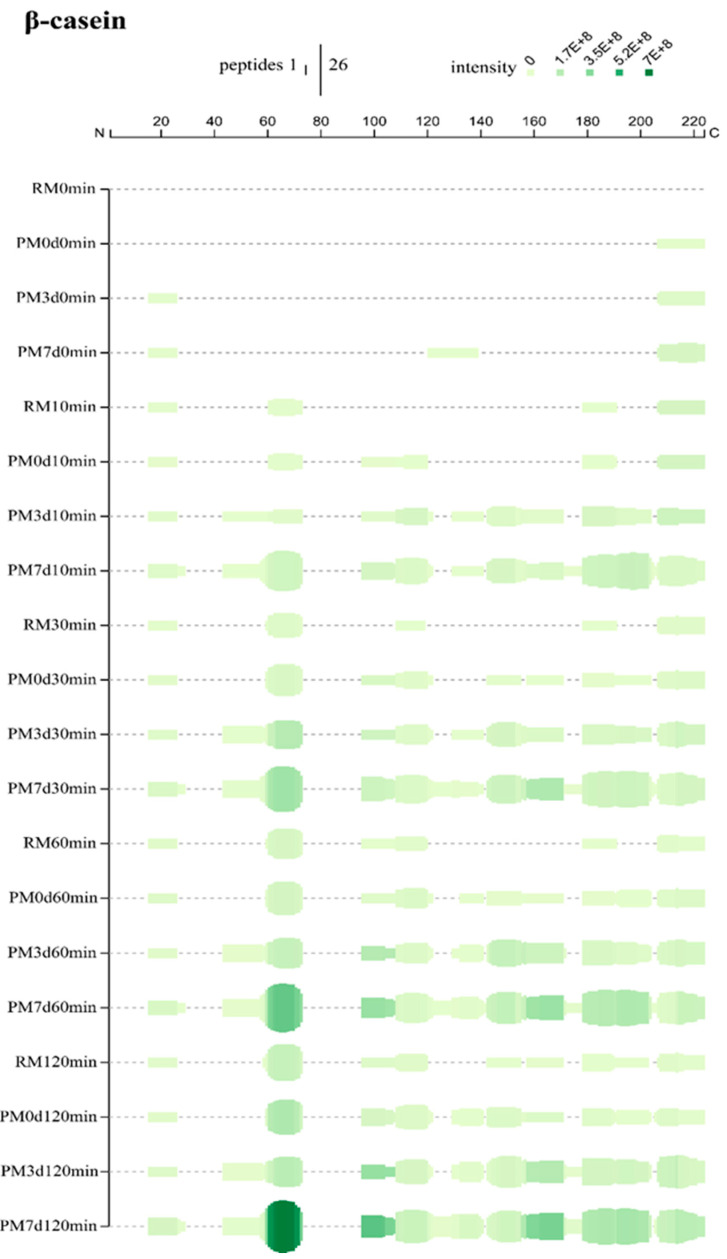
Peptide profiles originating from β-casein during the in vitro gastric digestion of raw milk (RM), pasteurized milk (PM0d), pasteurized milk upon 3 days of storage (PM3d), and pasteurized milk upon 7 days of storage (PM7d).

The occurrence of proteolysis in pasteurized milk during a long period of cold storage has been confirmed in our previous study [15]. The generated peptides caused by stored-induced proteolysis prior to digestion are shown in Table 1. To better understand the peptide generation mechanism, the peptides generated prior to digestion are also shown, along with peptide profiles during digestion (Figure 4, Figure 5 and Supplementary Materials Figure S5). All of the peptides originated from α_s1_-CN, α_s2_-CN, and β-CN. No peptide was found in κ-CN and β-Lg. Pasteurization, as a mild thermal processing technique, could induce two peptides (α_s1_-CN_200-214_ and β-CN_207-224_) generated. Proteolysis occurred in stored milk, especially in PM7d, which could change the protein structure and thereby alter the protein digestibility (such as the digestibility of γ-CN, which was different from that of β-CN). Moreover, the contents of the generated peptides were also altered during gastric digestion. For example, the intensity of peptide β-CN_208-221_ (YQEPVLGPVRGPFP) was similar between PM7d 0min and PM7d 10min and its intensity was gradually decreased with an increase in the digestion time. This result indicated that this peptide could not be formed during gastric digestion, whereas it could be further degraded under the action of pepsin.

### 3.6. Peptides Released during Digestion

**Figure 6:**
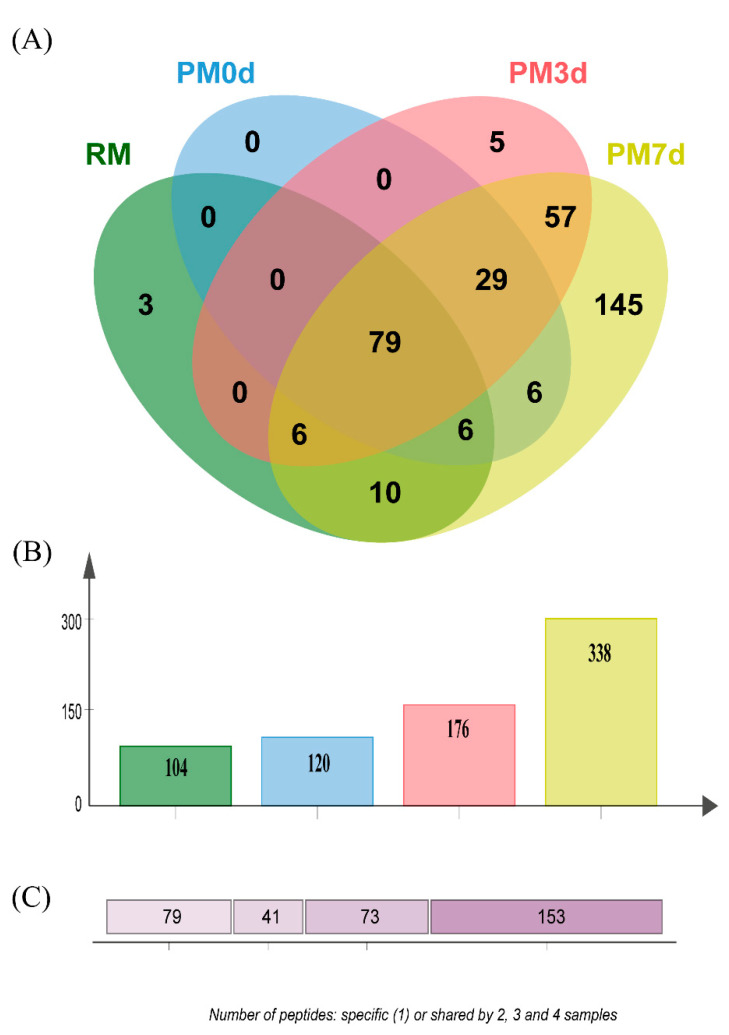
Venn diagram analysis of the identified peptides (**A**) from raw milk (RM), pasteurized milk (PM0d), pasteurized milk upon 3 days of storage (PM3d), and pasteurized milk upon 7 days of storage (PM7d) under in vitro digestion; (**B**) the number of peptides identified from each sample; (**C**) the number of specific (1) and shared peptides by 2, 3, and 4 samples.

The peptides released during the simulated gastric digestion were further analyzed using LC-MS/MS. A Venn diagram analysis of the identified peptides from each sample is shown in Figure 6. Three hundred and forty-six peptides in all of the samples during gastric digestion (the sum of all the time points of digestion) were identified through peptide identification. More peptides were found in pasteurized milk undergoing cold storage compared with those in raw and pasteurized milk (Figure 6B). The number of specific peptides (153 peptides only found in one sample) was higher than the number of shared peptides (73, 41, and 79 peptides shared by 2, 3, and 4 samples, respectively) (Figure 6C). The number of specific peptides found in the PM7d sample (145) was significantly higher than those in the RM (3), PM0d (0), and PM3d (5) sample (Figure 6A). Furthermore, a Venn diagram analysis of the peptides originating from the individual protein (α_s1_-CN, α_s2_-CN, β-CN, κ-CN, and β-Lg) in each sample under the whole process of in vitro digestion is shown in Supplementary Materials Figure S1. The visual difference performed using principal component analysis (PCA) further showed that the peptides in raw and pasteurized milk were close and revealed a significant difference between pasteurized milk during storage and raw/pasteurized milk at different digestion time points (Supplementary Materials Figure S2).

The peptides identified were matched with the primary structure of the major proteins (α_s1_-CN, α_s2_-CN, β-CN, κ-CN, and β-Lg) in milk to deduce the origins of and the differences in peptide profiles released upon pasteurization and storage at 10, 30, 60, and 120 min of digestion. Overall, the sequence coverages of the proteins upon processing and storage were increased with the time of digestion, despite a slight reduction of β-CN in PM7d at 120 min of digestion (Supplementary Materials Figure S3). Higher coverage rates of parent proteins in heated milk were more abundant compared to those in raw milk. A high number of peptides were found in stored milk compared to both raw and pasteurized milk at day 0.

Peptigram, an online dynamic visualization tool, provides a summary view of the peptide profiles of samples. The height of the green bars is proportional to the count of peptides overlapping this position. The intensity of the color (green) is proportional to the sum of the peptide intensities overlapping this position. More detailed peptide alignment maps are shown in Supplementary Materials Figure S3. In general, more peptides and higher peptide intensities were identified in stored milk compared to raw and pasteurized milk at day 0 (PM7d > PM3d > PM0d > RM) at the same digestion time point.

For α_s1_-casein, there was 60, 57, 62, and 70% sequence coverage for raw and pasteurized milk storage at 0, 3, and 7 d, respectively, at the end of gastric digestion (Supplementary Materials Figure S3A). At 10 min, only three regions (f16–37, f180–187, and f200–214) were hydrolyzed in raw milk, indicating that the hydrolysis began from the N-terminal and C-terminal regions of α_s1_-CN (Figure 4). New regions (f39–54 and f195–200) were found in pasteurized milk. The cleavage bonds Phe_38_-Phe_39_ and Phe_194_-Ser_195_, which were buried inside the hydrophobic core [1], were not easily accessible for pepsin. Heat treatment could provide exposure of the cleavage bonds, increasing their susceptibility to hydrolysis. No significant change was observed in PM3d compared with PM0d. However, more regions (95–110, 113–134, and 140–158) were found in PM7d at 10 min of gastric digestion, indicating that hydrolysis had started from the middle regions in pasteurized milk after 7 days of cold storage. More and higher intensities of peptides were found in PM7d samples. At 30 min, more peptides originating from new regions were identified from samples. The peptides from new regions (95–110 and 113–124) were identified in PM3d, indicating that the hydrolysis which started in the middle regions occurred in all of the pasteurized milk upon cold storage at 30 min of digestion. Comprehensive hydrolysis occurred in all of the samples after 120 min of digestion. The intensities of peptides from regions f169–179 and f195–211 in PM7d were significantly higher than those in other samples at the end of gastric digestion. In summary, more and higher intensities of peptides were identified from the regions (16–55, 95–110, and 195–210), which was consistent with previous works presenting peptidomics analyses of bovine and human milk protein-derived peptides during in vitro dynamic digestion or clinical trials [32,33]. Similar hydrolysis regions of α_s1_-CN were observed in all of the samples at the end of gastric digestion, although differences in peptide profiles existed in different samples during 120 min of digestion. More and higher intensities of peptides were found in PM7d samples compared with other samples.

β-casein, mostly present in the interior of casein micelles, is the most hydrophobic of all caseins [1]. At 10 min, most of the peptides were from the regions between residues R_16_-E_26_ (N-terminal), Q_61_-L_73_, S_179_-K_191_, and G_214_-V_224_ (C-terminal) in raw milk, indicating that the hydrolysis started from this part of β-CN (Figure 5). Pasteurization produced new hydrolysis from the region 96–120. More hydrolysis regions were detected in pasteurized milk upon cold storage. The coverage patterns were close between PM3d and PM7d samples, while more and higher intensities of peptides were found in PM7d. Similar phenomena were also found at 30, 60, and 120 min of gastric digestion. At the end of digestion, there was 50, 57, 68, and 74% sequence coverage for RM, PM0d, PM3d, and PM7d, respectively (Supplementary Materials Figure S3B). The peptides were the most numerous in PM7d compared to other samples. Moreover, the intensities of peptides in PM7d were significantly higher than the ones in other samples, especially the peptides from L_60_-L_73_. The peptides from the region K_44_-L_60_ were only identified in pasteurized milk upon storage, indicating that cold storage can make this region accessible to hydrolysis. The refrigeration of milk can induce the reversible release of β-CN from the micelle into the serum phase [5]. Our previous study showed ~20% β-CN dissociation from the casein micelles in pasteurized milk upon cooling [15]. The dissociation of β-CN would cause a slight loosening of the casein micelle’s structure, which explained the increase in the micelle size of ~14–16 nm observed after 1 day of cold storage of the pasteurized milk [15]. The loose structure of the casein micelle that formed in pasteurized milk upon cold storage could increase the availability of caseins for pepsinolysis. Moreover, β-CN in the serum may be easier to pepsin hydrolyze compared to that in the casein micelle.

Low number of peptides were identified in κ-CN compared to α_s1_-CN and β-CN (Supplementary Materials Figure S5A). These results were possibly due to the fact that lactosylation, glycosylation, and protein-protein interaction can increase the difficulty of κ-CN peptide sequencing [11]. Additionally, κ-CN is about three times less abundant than α_s1_-CN and β-CN [34]. In the initial 10 min of gastric digestion, most of the peptides originating from the middle regions 54–71, 88–96, and 117–126 were identified in raw milk. The number of peptides found in pasteurized milk (11 peptides) was higher than that in raw milk (7 peptides) and six peptides were common in both. Peptides from the C-terminal region 180–190 were found in PM3d and PM7d at 10 min of digestion, while in RM and PM0d, they were found at 30 min or more. The results indicated that cold storage could make this region easily accessible to hydrolysis. There was a minor change in peptide profiles in all of the samples at 30 and 60 min of digestion. The peptides from the new region 146–167 were found in all of the samples after 30 min of gastric digestion. The peptides from the regions 39–50 and 127–145 were identified in all of the samples after 120 min of digestion, although these peptides were found in PM7d during the whole process of gastric digestion. Overall, κ-CN exhibited rapid hydrolysis at the initial stage of digestion and minor changes during subsequent digestion. Several regions (22–38, 98–114, and 168–179) were found to be resistant to hydrolysis in all of the samples during digestion, suggesting that these regions were resistant to pepsin hydrolysis.

Low sequence coverage and a low number of peptides identified in α_s2_-CN resulted from the low content of α_s2_-CN in milk (Supplementary Materials Figure S5B). At the initial 10 min of digestion, several peptides were found in PM0d and PM3d (1 and 3 peptides in PM0d and PM3d, respectively), whereas no peptide was found in raw milk. Relatively more peptides were identified in PM7d. At 30 min of digestion, the peptides originating from the middle regions (96–113 and 130–138) were identified in raw milk, indicating that the hydrolysis started from these regions. More peptides were found along with the gastric digestion time. In general, the impact of heat treatment on the peptide profile of α_s2_-CN was limited (only one peptide ALPQYLKT was generated from PM0d at 10 min). A long period of cold storage could induce the generation of more peptides. Still, many regions (such as N-terminal, 139–161) were resistant to pepsin hydrolysis.

Low numbers of peptides from β-Lg were detected in raw milk during gastric digestion (0, 1, 2, and 5 peptides at 10, 30, 60, and 120 min of digestion, Supplementary Materials Figure S5C), which was consistent with native β-Lg’s resistance to pepsin hydrolysis [35]. The peptides from the regions 28–44, 58–70, and 112–120 were detected in PM0d at 10 min, indicating that pasteurization induced conformational changes in β-Lg and thus increased the susceptibility to pepsin action [14]. No new peptides were found in PM0d at 30 min, indicating that the hydrolysis of β-Lg in heated milk mainly occurred during the initial period of gastric digestion. Peram (7) reported that the high-molecular-weight aggregates formed by denatured β-Lg (e.g., pentamers, tetramers, and trimers) were digested rapidly, whereas native β-Lg and dimers were resistant to pepsin hydrolysis. Therefore, the hydrolysis of β-Lg in pasteurized milk during the initial period of digestion possibly resulted from the hydrolysis of high-molecular-weight aggregates. More peptides originating from new regions (i.e., 17–27, 48–58, 91–98, 99–111, and 139–165) were identified in pasteurized milk upon cold storage, especially in pasteurized milk after 7 days of storage. These results demonstrated a rapid hydrolysis of β-Lg in stored milk. Although the mechanism behind this phenomenon is not clear, it is probably due to the fact that the interaction of β-Lg and κ-CN and re-association of β-Lg/κ-CN complexes with micelles during storage might change the structure and phase distribution of β-Lg, increasing pepsin’s accessibility to potential cleavage sites.

Table 2 shows the identified bioactive peptides and their potential biological activity matched to the BIOPEP database in each sample at the end (120 min) of gastric digestion. Twenty-one peptides were matched to the database and the majority of these peptides had angiotensin-converting enzyme (ACE) inhibitory (52%) and antimicrobial (29%) functionalities; other bioactivities included immunomodulatory (19%), DPP-IV inhibitory (14%), and antithrombin (5%) bioactivities. The bioactive peptides displayed different qualitative and quantitative results in each sample. For instance, the ACE-inhibitory peptide from α_s1_-CN f172–179 (DAYPSGAW) was found in all of the samples. However, the content of this peptide in raw and pasteurized milk was higher than that in pasteurized milk upon storage. Therefore, processing and storage played decisive roles in bioactive peptide generation.

### 3.7. Amino Acids Released during Digestion

Understanding the pattern of amino acids during gastric digestion is an important step for better investigating milk protein metabolism, which can determine their availability and absorption in the intestinal tract. Most of the amino acids in all of the samples increased during gastric digestion (Table 3). The high level of Phe and Leu present in all of the samples at the end of digestion was due to the fact that pepsin preferentially cleaves the C-terminal end of Phe and Leu [36]. The levels of the majority of free amino acids in stored pasteurized milk were higher than pasteurized milk and those in pasteurized milk were higher than raw milk at 120 min, except for Lys. This might be due to the fact that glycation and lactosylation modify the side chains of proteins, inducing the blocking of Lys residues to hydrolysis [37]. In general, the higher levels of amino acids found in PM7d indicated that rapid hydrolysis occurred in pasteurized milk upon cold storage, which was consistent with the results of LC-MSMS.

## 4. Conclusions

The coagulation behavior of milk and protein digestibility were investigated in pasteurized milk upon storage under dynamic in vitro gastric digestion. A high level of hydration in curds formed in pasteurized milk upon 7-day cold storage compared to raw and pasteurized milk indicated that pepsin could easily enter the interior of curds, increasing the digestion rate. The results obtained from OPA, SDS-PAGE, LC-MSMS, and amino acid analysis further revealed that rapid hydrolysis of milk proteins occurred in pasteurized milk upon cold storage. Higher intensities of peptides were found in pasteurized milk upon subsequent storage. The peptide patterns in different samples at different digestion times were different, which indicated that the protein digestibility was altered upon pasteurization and cold storage. Moreover, heat treatment and storage played decisive roles in bioactive peptide generation. This study can provide insights into and directions for the storage of pasteurized milk for further clinical studies to assess whether the release of bioactive peptides at specific time points during digestion could yield a modified physiological response, thereby resulting in desired health outcomes. Further research is needed to determine site-specific protein modifications during storage and their relationship to in vitro and in vivo protein digestibility.

## Figures and Tables

**Table 1:** Peptides identified in raw milk (RM), pasteurized milk (PM0d), pasteurized milk upon 3 days of storage (PM3d), and pasteurized milk upon 7 days of storage (PM7d) prior to digestion.

Parent Protein	Peptide Sequence	Position	RM	PM0d	PM3d	PM7d
α_s1_-CN	RPKHPIKHQGLPQEVLNENLLR	16–37				√
	SDIPNPIGSENSEKTTMPLW	195–214			√	√
	PIGSENSEKTTMPLW	200–214		√	√	√
	SEKTTMPLW	206–214				√
α_s2_-CN	TKVIPYVRYL	213–222				√
β-CN	RELEELNVPGE	16–26			√	√
	HKEMPFPKYPVEPFTESQS	121–139				√
	LYQEPVLGPVRGPFPIIV	207–224		√	√	√
	YQEPVLGPVRGPFP	208–221				√
	YQEPVLGPVRGPFPIIV	208–224			√	√
	PVRGPFPIIV	215–224				√

**Table 2:** Bioactive peptides identified after the gastric digestion of raw milk (RM), pasteurized milk (PM0d), and pasteurized milk during storage (PM3d and PM7d) with the BIOPEP database, and the digestive time (min) at which they were identified in each sample.

Protein	Sequence	Residues	Bioactivity	References ^1^	Samples ^2^			
					RM	PM0d	PM3d	PM7d
*α_s1_-CN*	FVAPFPEVFG	39–48	ACE-inhibitory	[1]	1.82	0.00	1.69	1.00
	LRLKKYKVPQL	114–124	Antimicrobial	[2,3]	3.19	1.00	3.67	3.12
	LAYFYPEL	157–164	Immunomodulatory	[4]	77.44	5.32	2.10	1.00
	DAYPSGAW	172–179	ACE-inhibitory	[5]	12.64	11.76	8.48	1.00
	SDIPNPIGSENSEK	195–208	Antimicrobial	[6]	1.00	2.69	1.47	1.95
*β-CN*	VENLHLPLPLL	145–155	ACE-inhibitory	[1]	0.00	0.00	10.86	1.00
	NLHLPLPLL	147–155	ACE-inhibitory	[1]	0.00	0.00	1.53	1.00
	LYQEPVLGPVRGPFPIIV	207–224	Immunomodulatory	[7]	0.00	0.00	1.00	0.00
	YQEPVLGPVR	208–217	ACE-inhibitory	[8]	0.00	0.00	2.97	1.00
			Immunomodulatory	[9]				
	YQEPVLGPVRG	208–218	ACE-inhibitory	[10]	0.00	0.00	4.36	1.00
	YQEPVLGPVRGPFPIIV	208–224	Immunomodulatory	[11]	3.52	5.75	2.78	1.00
			antithrombin	[12]				
			Antimicrobial	[13]				
			ACE-inhibitory	[14]				
*κ-CN*	YYQQKPVA	63–70	Antimicrobial	[15]	1.00	1.05	1.66	2.33
	HPHPHLSF	119–126	ACE-inhibitory	[16]	0.00	0.00	2.06	1.00
	MAIPPKKNQDKTEIPTINT	127–145	Antimicrobial	[17]	1.00	0.00	0.00	0.00
	VESTVATL	160–167	Antimicrobial	[15]	1.01	1.00	1.36	4.27
*β-Lg*	LIVTQTMK	17–24	Cytotoxic	[18]	0.00	0.00	0.00	1.00
	LDIQKVAGTW	26–35	ACE-inhibitory	[19]	0.00	1.00	0.00	27.83
	IQKVAGTW	28–35	DPP-IV Inhibitory	[19]	0.00	1.21	2.56	6.49
			ACE-inhibitory	[19]				
	DAQSAPLRVY	49–58	ACE-inhibitory	[20,21]	0.00	0.00	0.00	1.00
	LKPTPEGDL	62–70	DPP-IV Inhibitory	[22]	0.00	0.00	0.00	1.00
	LKPTPEGDLE	42–71	DPP-IV Inhibitory	[22]	0.00	0.00	0.00	1.00

^1^ References are listed in Supplementary Materials Table S1. ^2^ The intensities of the specific bioactive peptide in different samples are normalized to the sample which has the lowest intensity of the peptide. 0.00 means not detected.

**Table 3:** Composition and content (mg/100 g) of free amino acids released from raw milk (RM), pasteurized milk (PM0d), pasteurized milk upon 3 days of storage (PM3d), and pasteurized milk upon 7 days of storage (PM7d) at 30, 60, and 120 min of gastric in vitro digestion.

Amino Acid	Amino Acid Released during Digestion											
	30 min				60 min				120 min			
	RM	PM0d	PM3d	PM7d	RM	PM0d	PM3d	PM7d	RM	PM0d	PM3d	PM7d
Essential amino acid												
Thr	0.1 ± 0 ^aA^	0.1 ± 0.1 ^aA^	0.3 ± 0.1 ^aA^	0.8 ± 0.1 ^bA^	0.2 ± 0.1 ^aA^	0.2 ± 0.1 ^aA^	0.3 ± 0.1 ^aA^	2.6 ± 0.2 ^bB^	0.6 ± 0.1 ^aB^	1.2 ± 0.2 ^bB^	2.7 ± 0.3 ^cB^	4.6 ± 0.1 ^dC^
Cys	0.2 ± 0.1 ^aA^	0.4 ± 0.2 ^aA^	0.6 ± 0.1 ^aA^	1.4 ± 0.2 ^bA^	0.3 ± 0.1 ^aA^	0.3 ± 0.2 ^aA^	0.4 ± 0.2 ^aA^	1.9 ± 0.2 ^bB^	0.6 ± 0.2 ^aB^	0.8 ± 0.3 ^bB^	1.5 ± 0.1 ^cB^	2.8 ± 0.3 ^dC^
Lys	0.4 ± 0.3 ^aA^	0.4 ± 0.1 ^aA^	0.6 ± 0.2 ^aA^	1.6 ± 0.2 ^bA^	2.7 ± 0.1 ^aB^	1.4 ± 0.1 ^bB^	1.3 ± 0.1 ^bB^	3.6 ± 0.2 ^cB^	5.9 ± 0.3 ^aC^	2.6 ± 0.2 ^bC^	3.6 ± 0.2 ^aC^	3.5 ± 0.3 ^cB^
Met	0.5 ± 0.3 ^aA^	0.5 ± 0.2 ^aA^	0.7 ± 0.2 ^aA^	0.8 ± 0.1 ^aA^	0.6 ± 0.1 ^aA^	0.6 ± 0.2 ^aA^	0.7 ± 0.2 ^aA^	1.3 ± 0.1 ^bB^	0.8 ± 0.1 ^aA^	1.1 ± 0.2 ^bB^	2.6 ± 0.1 ^bC^	5.7 ± 0.2 ^cC^
Phe	1.9 ± 0.2 ^aA^	2.1 ± 0 ^abA^	2.3 ± 0.1 ^bA^	2.7 ± 0.3 ^cA^	3.5 ± 0.2 ^aB^	4.4 ± 0.2 ^bB^	5.1 ± 0.1 ^cB^	8.2 ± 0.2 ^dB^	9.6 ± 0.2 ^aC^	11.1 ± 0.2 ^bC^	11.5 ± 0.3 ^bC^	11.5 ± 0.4 ^bC^
Ile	0.1 ± 0.1 ^aA^	0.1 ± 0 ^aA^	0.3 ± 0.1 ^bA^	0.4 ± 0.1 ^bA^	0.2 ± 0.1 ^aA^	0.4 ± 0.1 ^bB^	0.4 ± 0 ^bA^	0.9 ± 0.1 ^cB^	0.3 ± 0.1 ^aA^	0.9 ± 0.1 ^bC^	2.2 ± 0.2 ^cB^	3.3 ± 0.2 ^dC^
Leu	0.8 ± 0.1 ^aA^	1.1 ± 0.2 ^abA^	1.3 ± 0.1 ^bA^	1.8 ± 0.2 ^aA^	2.0 ± 0.2 ^aB^	2.4 ± 0.2 ^aB^	3.0 ± 0.1 ^cB^	5.1 ± 0.2 ^dB^	6.6 ± 0.2 ^aC^	7.4 ± 0.3 ^bB^	8.1 ± 0.2 ^cB^	8.7 ± 0.3 ^dC^
His	0.1 ± 0 ^aA^	0.1 ± 0 ^aA^	0.1 ± 0 ^aA^	0.1 ± 0 ^aA^	0.2 ± 0.2 ^aA^	0.1 ± 0 ^aA^	0.3 ± 0.2 ^aA^	0.6 ± 0.1 ^bB^	0.4 ± 0.1 ^aB^	2.3 ± 0.2 ^aB^	2.9 ± 0.1 ^cB^	4.1 ± 0.2 ^dC^
Tyr	0.6 ± 0.1 ^aA^	0.6 ± 0.1 ^aA^	0.7 ± 0.1 ^aA^	3.2 ± 0.1 ^bA^	0.6 ± 0.1 ^aA^	0.6 ± 0.1 ^aA^	0.9 ± 0 ^bA^	2.6 ± 0.1 ^cB^	0.8 ± 0.1 ^aA^	1.4 ± 0.2 ^bB^	3.2 ± 0.3 ^cB^	5.1 ± 0.5 ^dC^
Val	0.9 ± 0.1 ^aA^	1.1 ± 0.1 ^aA^	1.0 ± 0.1 ^aA^	1.7 ± 0.2 ^bA^	0.9 ± 0.1 ^aA^	0.9 ± 0.1 ^aA^	1.1 ± 0.2 ^aA^	2.7 ± 0.2 ^bB^	1.1 ± 0.2 ^aA^	1.9 ± 0.1 ^bB^	2.7 ± 0.2 ^cB^	4.4 ± 0.5 ^dC^
Non-essential amino acid												
Ser	0.1 ± 0 ^aA^	0.1 ± 0 ^aA^	0.2 ± 0 ^aA^	0.4 ± 0.1 ^bA^	0.4 ± 0.1 ^aB^	0.4 ± 0.1 ^aA^	0.4 ± 0.1 ^aA^	0.9 ± 0.1 ^bB^	0.7 ± 0.2 ^aC^	1.4 ± 0.2 ^bB^	1.7 ± 0.3 ^bB^	3.2 ± 0.2 ^cC^
Arg	0.5 ± 0.1 ^aA^	0.6 ± 0.1 ^aA^	0.9 ± 0.2 ^aB^	1.2 ± 0.2 ^cA^	0.8 ± 0.1 ^aA^	1.1 ± 0.2 ^bA^	1.1 ± 0.2 ^aA^	3.6 ± 0.2 ^bB^	1.6 ± 0.1 ^aA^	2.2 ± 0.1 ^bB^	2.4 ± 0.2 ^bB^	6.0 ± 0.4 ^cC^
Asp	0.2 ± 0.1 ^aA^	0.2 ± 0.1 ^aA^	0.3 ± 0.2 ^aA^	0.5 ± 0.1 ^bA^	0.3 ± 0.3 ^aA^	0.5 ± 0.2 ^aA^	0.6 ± 0.3 ^aB^	0.8 ± 0.2 ^aB^	0.4 ± 0.2 ^aA^	0.8 ± 0.2 ^bA^	0.8 ± 0.2 ^bB^	1.4 ± 0.00 ^cC^
Gly	0.6 ± 0.1 ^aA^	0.6 ± 0.2 ^aA^	0.8 ± 0.2 ^aA^	0.9 ± 0.2 ^aA^	0.7 ± 0.2 ^aA^	0.9 ± 0.2 ^aA^	0.9 ± 0.2 ^aA^	1.2 ± 0.1 ^aB^	0.9 ± 0.2 ^aA^	1.4 ± 0.2 ^bC^	1.6 ± 0.1 ^bB^	2.4 ± 0.2 ^cC^
Glu	5.4 ± 0.1 ^aA^	5.4 ± 0.1 ^aA^	5.7 ± 0.2 ^aA^	6.8 ± 0.2 ^bA^	5.5 ± 0.2 ^aA^	5.7 ± 0.2 ^aA^	6.0 ± 0.2 ^aA^	7.8 ± 0.2 ^bB^	6.4 ± 0.2 ^aB^	8.0 ± 0.2 ^bB^	8.5 ± 0.1 ^cB^	9.2 ± 0.1 ^dC^
Ala	0.4 ± 0.1 ^aA^	0.4 ± 0.1 ^aA^	0.5 ± 0.2 ^aA^	0.7 ± 0.1 ^aA^	0.5 ± 0.1 ^aA^	0.5 ± 0.1 ^aA^	0.6 ± 0.2 ^aA^	1.7 ± 0.2 ^bB^	0.7 ± 0.2 ^aA^	1.5 ± 0.2 ^bB^	3.5 ± 0.2 ^cB^	4.5 ± 0.2 ^dC^
Pro	0.5 ± 0.1 ^aA^	0.5 ± 0.1 ^aA^	0.6 ± 0.2 ^aA^	0.9 ± 0.2 ^bA^	0.7 ± 0.2 ^aA^	0.7 ± 0.2 ^aA^	0.7 ± 0.1 ^aA^	1.1 ± 0.1 ^bA^	1.1 ± 0.1 ^aA^	1.8 ± 0.1 ^bB^	2.3 ± 0.2 ^cB^	2.6 ± 0.2 ^dB^
Total	13.4 ^aA^	14.1 ^aA^	17.1 ^bA^	25.7 ^cA^	22.4 ^aB^	21.8 ^aB^	23.2 ^aB^	47.4 ^bB^	38.5 ^aC^	47.8 ^bC^	62.4 ^cC^	86.2 ^dC^

Data shown (mean ± standard deviation) are the mean values of three independent samples and each was measured in triplicate. Values in a row with different lowercase superscripts (a–c) F differ significantly (*p* < 0.05) in different samples at the same digestion time. Values in a row with different uppercase superscripts (A–C) differ significantly (*p* < 0.05) in each sample at different digestion times.

## Supplementary Materials

The following are available online at https://www.mdpi.com/2304-8158/9/8/998/s1. Table S1: List of references for the bioactive peptides in Table 1; Figure S1: Venn diagram analysis of the identified peptides from α_s1_-casein (A), α_s2_-casein (B), β-casein (C), κ-casein (D), and β-Lg (E) in raw milk (RM), pasteurized milk (PM0d), pasteurized milk upon 3 days of storage (PM3d), and pasteurized milk upon 7 days of storage (PM7d) during the whole in vitro digestion process; Figure S2. PCA score plots of the identified peptides from raw milk (RM), pasteurized milk (PM0d), pasteurized milk upon 3 days of storage (PM3d), and pasteurized milk upon 7 days of storage (PM7d) at 10, 30, 60, and 120 min of in vitro gastric digestion; Figure S3: Peptide coverages of parent protein sequences (A: α_S1_-casein, B: β-casein, C: κ-casein, D: α_S2_-casein, and E: β-lactoglobulin) from raw milk (RM), pasteurized milk (PM0d), pasteurized milk upon 3 days of storage (PM3d), and pasteurized milk upon 7 days of storage (PM7d) at 10, 30, 60, and 120 min of digestion; Figure S4: Peptide alignment maps originating from α_s1_-casein, α_s2_-casein, β-casein, κ-casein, and β-lactoglobulin (β-lg) during the in vitro gastric digestion of raw milk (RM), pasteurized milk (PM0d), pasteurized milk upon 3 days of storage (PM3d), and pasteurized milk upon 7 days of storage (PM7d); Figure S5: Peptide profiles originating from κ-casein (A), α_s2_-casein (B), and β-lactoglobulin (β-lg, C) during the in vitro gastric digestion of raw milk (RM), pasteurized milk (PM0d), pasteurized milk upon 3 days of storage (PM3d), and pasteurized milk upon 7 days of storage (PM7d).
