# Supplementary material for: Influence of Pasteurization and Storage on Dynamic In Vitro Gastric Digestion of Milk Proteins: Quantitative Insights Based on Peptidomics
Source: Foods. 2020 Jul 25;9(8):998. doi: 10.3390/foods9080998 (PMC7466191; doi:10.3390/foods9080998)
Supplement: Supplementary file 1 [file foods-09-00998-s001.pdf]

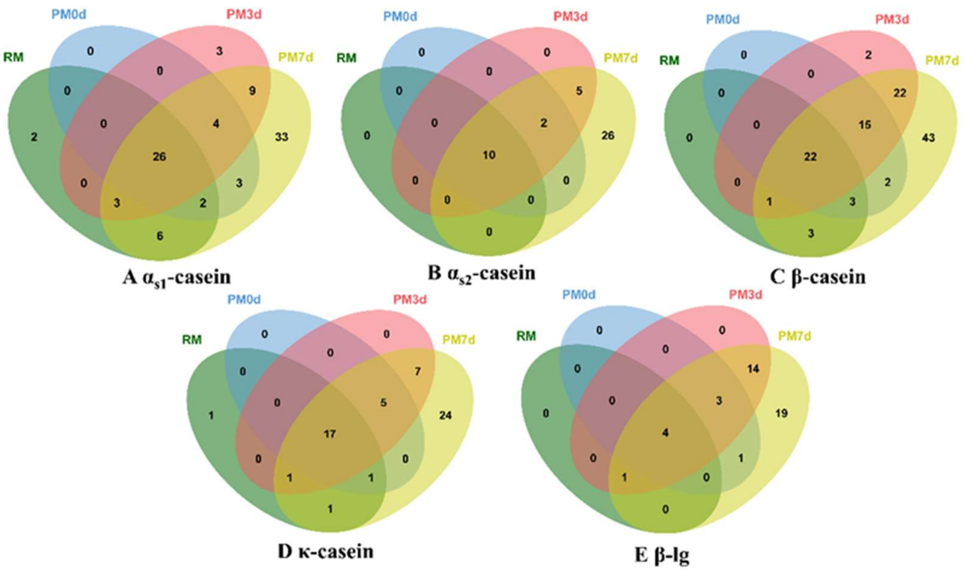

Figure S1. Venn diagram analysis of the identified peptides from αs1-casein (A), αs2-casein (B), β-casein (C), κ-casein (D) and β-Lg (E) in raw milk (RM), pasteurized milk (PM0d), pasteurized milk upon 3 days storage (PM3d) and pasteurized milk upon 7 days storage (PM7d) under the whole in vitro digestion.

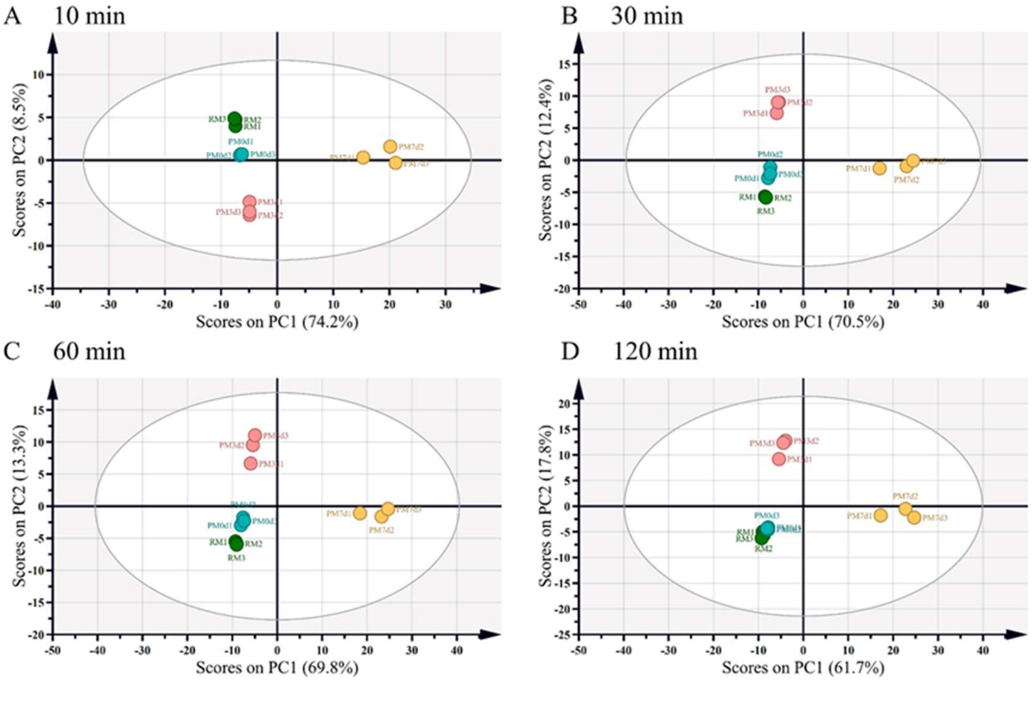

Figure S2. PCA score plots of the identified peptides from raw milk (RM), pasteurized milk (PM0d), pasteurized milk upon 3 days storage (PM3d) and pasteurized milk upon 7 days storage (PM7d) at 10, 30, 60 and 120 min of the in vitro gastric digestion.

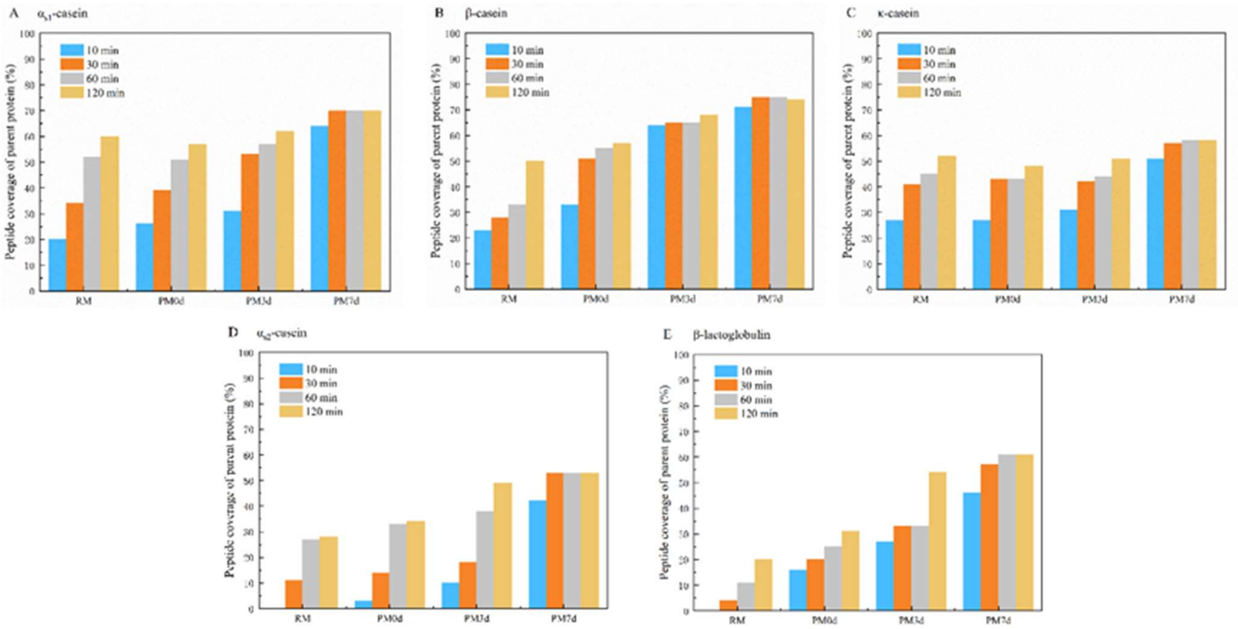

Figure S3. Peptide coverages of parent protein sequences (A: α_{S1}-casein, B: β-casein, C: κ-casein, D: α_{S2}-casein and E: β-lactoglobulin) from raw milk (RM), pasteurized milk (PM0d), pasteurized milk upon 3 days storage (PM3d) and pasteurized milk upon 7 days storage (PM7d) at 10, 30, 60 and 120 min of digestion.

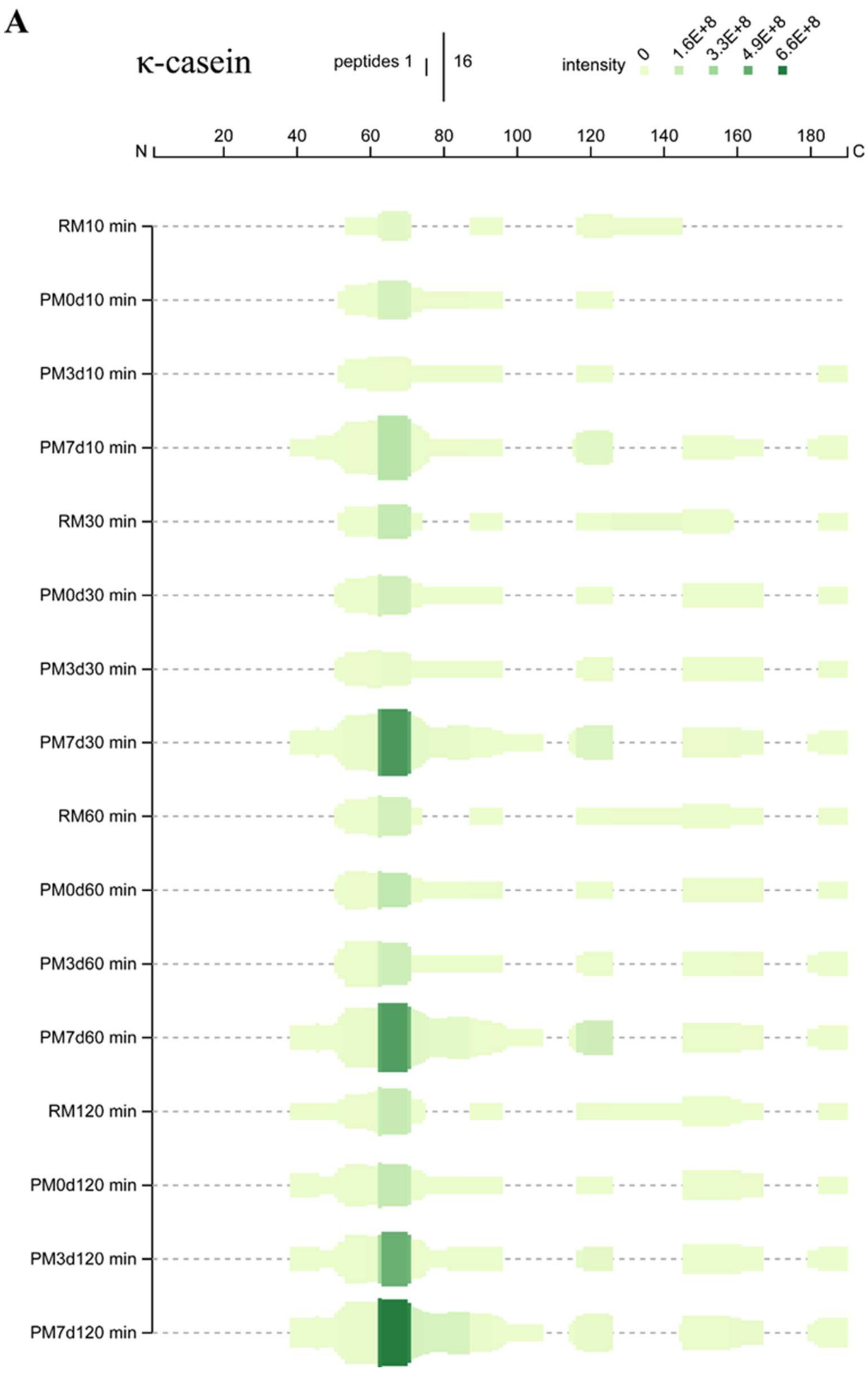

A
κ-casein
peptides 1
16
intensity
0
1.6E+8
3.3E+8
4.9E+8
6.6E+8
N
20
40
60
80
100
120
140
160
180
C
RM10 min
PM0d10 min
PM3d10 min
PM7d10 min
RM30 min
PM0d30 min
PM3d30 min
PM7d30 min
RM60 min
PM0d60 min
PM3d60 min
PM7d60 min
RM120 min
PM0d120 min
PM3d120 min
PM7d120 min

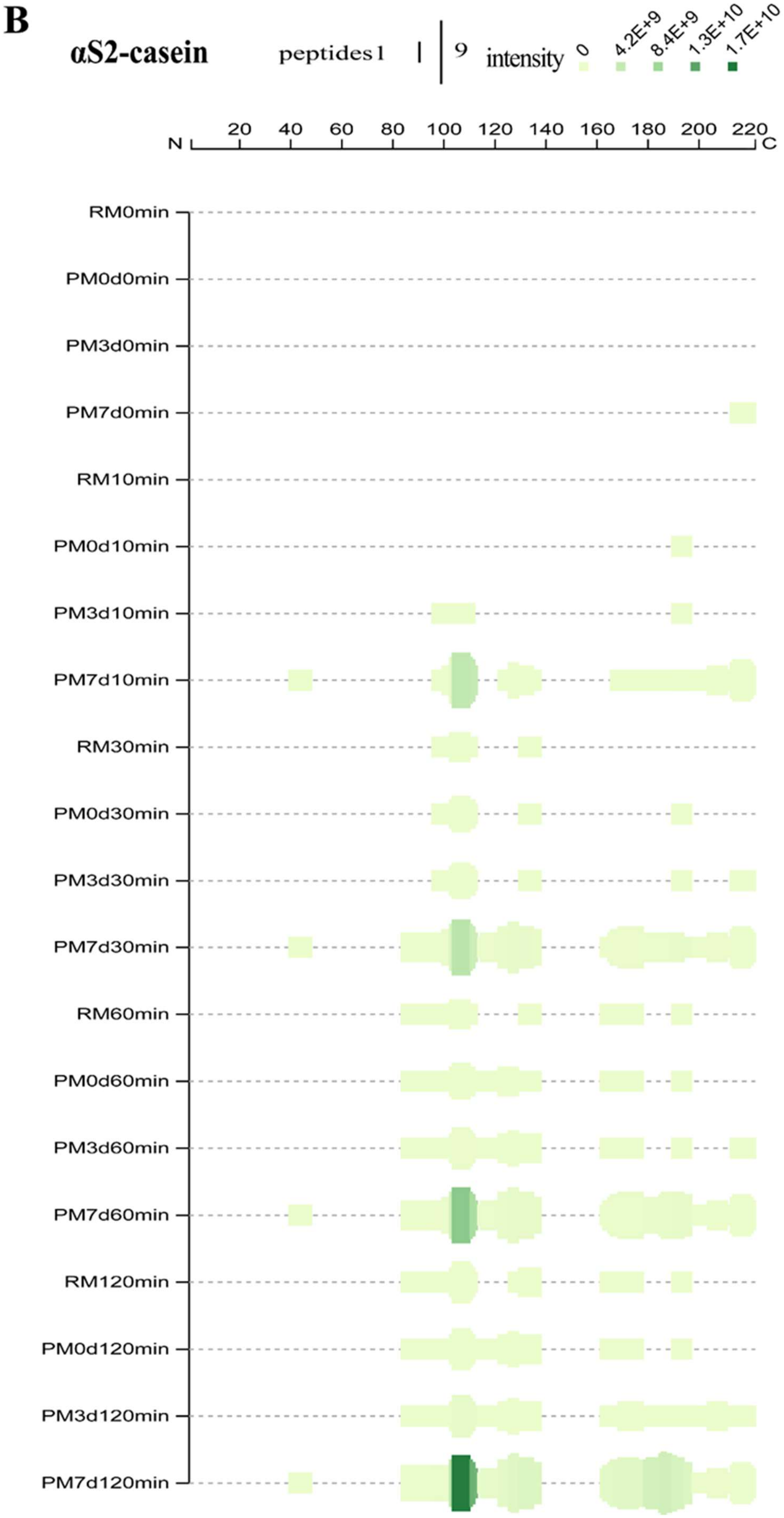

B
αS2-casein
peptides 1
9
intensity
0
4.2E+9
8.4E+9
1.3E+10
1.7E+10
N
20
40
60
80
100
120
140
160
180
200
220
C
RM0min
PM0d0min
PM3d0min
PM7d0min
RM10min
PM0d10min
PM3d10min
PM7d10min
RM30min
PM0d30min
PM3d30min
PM7d30min
RM60min
PM0d60min
PM3d60min
PM7d60min
RM120min
PM0d120min
PM3d120min
PM7d120min

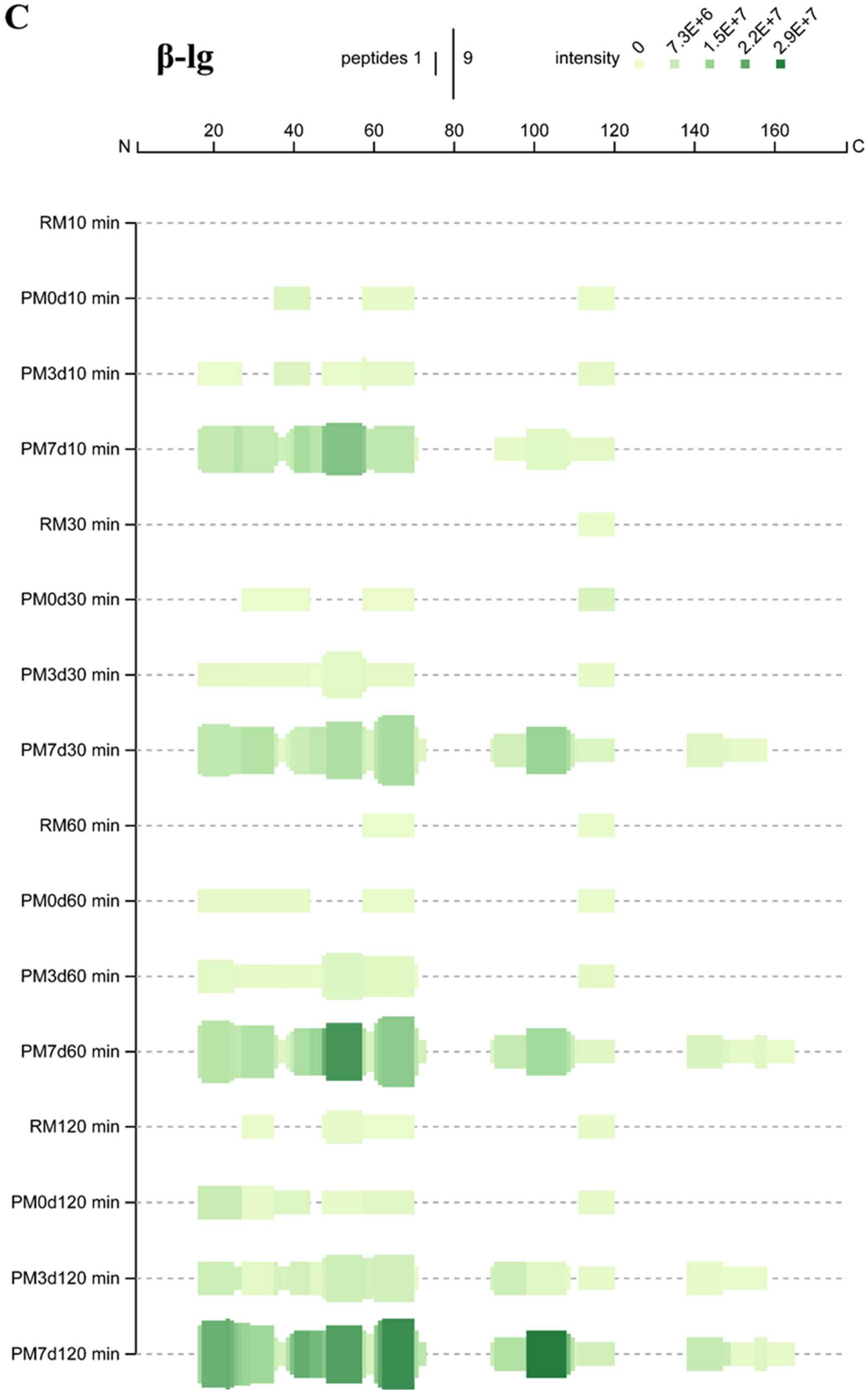

Figure S5. Peptide profiles originating from κ-casein (A), α_{s2}-casein (B) and β-lactoglobulin (β-lg, C) during in vitro gastric digestion of raw milk (RM), pasteurized milk (PM0d), pasteurized milk upon 3 days storage (PM3d) and pasteurized milk upon 7 days storage (PM7d).

Table 1. List of references for the bioactive peptides in Table 1.

Ref No.	Reference
1	Robert, M. C., Razaname, A., Mutter, M., & Juillerat, M. A. (2004). Identification of angiotensin-I-converting enzyme inhibitory peptides derived from sodium caseinate hydrolysates produced by Lactobacillus helveticus NCC 2765. Journal of Agricultural and Food Chemistry, 52(23), 6923-6931.
2	McCann, K. B., Shiell, B. J., Michalski, W. P., Lee, A., Wan, J., Roginski, H., & Coventry, M. J. (2006). Isolation and characterisation of a novel antibacterial peptide from bovine αS1-casein. International Dairy Journal, 16(4), 316-323.
3	Tang, W., Yuan, H., Zhang, H., Wang, L., Qian, H., & Qi, X. (2015). An antimicrobial peptide screened from casein hydrolyzate by Saccharomyces cerevisiae cell membrane affinity method. Food control, 50, 413-422.
4	Totsuka, M., Kakehi, M., Kohyama, M., Hachimura, S., Hisatsune, T., & Kaminogawa, S. (1998). Enhancement of antigen-specific IFN-γ production from CD8+ T cells by a single amino acid-substituted peptide derived from bovine αs1-casein. Clinical immunology and immunopathology, 88(3), 277-286.
5	Pihlanto-Leppälä, A., Rokka, T., & Korhonen, H. (1998). Angiotensin I converting enzyme inhibitory peptides derived from bovine milk proteins. International Dairy Journal, 8(4), 325-331.
6	Hayes, M., Ross, R. P., Fitzgerald, G. F., Hill, C., & Stanton, C. (2006). Casein-derived antimicrobial peptides generated by Lactobacillus acidophilus DPC6026. Appl. Environ. Microbiol., 72(3), 2260-2264.
7	Coste, M., Rochet, V., Léonil, J., Mollé, D., Bouhallab, S., & Tomé, D. (1992). Identification of C-terminal peptides of bovine β-casein that enhance proliferation of rat lymphocytes. Immunology Letters, 33(1), 41-46.
8	Lu, Y., Govindasamy-Lucey, S., & Lucey, J. A. (2016). Angiotensin-I-converting enzyme-inhibitory peptides in commercial Wisconsin Cheddar cheeses of different ages. Journal of dairy science, 99(1), 41-52.
9	Kayser, H., & Meisel, H. (1996). Stimulation of human peripheral blood lymphocytes by bioactive peptides derived from bovine milk proteins. FEBS letters, 383(1-2), 18-20.
10	Sagardia, I., Iloro, I., Elortza, F., & Bald, C. (2013). Quantitative structure–activity relationship based screening of bioactive peptides identified in ripened cheese. International Dairy Journal, 33(2), 184-190.
11	Sandré, C., Gleizes, A., Forestier, F., Gorges-Kergot, R., Chilmonczyk, S., Léonil, J., ... & Labarre, C. (2001). A peptide derived from bovine β-casein modulates functional properties of bone marrow-derived macrophages from germfree and human flora-associated mice. The Journal of nutrition, 131(11), 2936-2942.
12	Rojas-Ronquillo, R., Cruz-Guerrero, A., Flores-Nájera, A., Rodríguez-Serrano, G., Gómez-Ruiz, L., Reyes-Grajeda, J. P., ... & García-Garibay, M. (2012). Antithrombotic and angiotensin-converting enzyme inhibitory properties of peptides released from bovine casein by Lactobacillus casei Shirota. International Dairy Journal, 26(2), 147-154.
13	Birkemo, G. A., O'Sullivan, O., Ross, R. P., & Hill, C. (2009). Antimicrobial activity of two peptides casecidin 15 and 17, found naturally in bovine colostrum. Journal of applied microbiology, 106(1), 233-240.
14	Yamamoto, N., Akino, A., & Takano, T. (1994). Antihypertensive effect of the peptides derived from casein by an extracellular proteinase from Lactobacillus helveticus CP790. Journal of dairy science, 77(4), 917-922.
15	Lopez-Exposito, I., Minervini, F., Amigo, L., & Recio, I. (2006). Identification of antibacterial peptides from bovine κ-casein. Journal of food protection, 69(12), 2992-2997.
16	Miguel, M., Gómez-Ruiz, J. Á., Recio, I., & Aleixandre, A. (2010). Changes in arterial blood pressure after single oral administration of milk-casein-derived peptides in spontaneously hypertensive rats. Molecular nutrition & food research, 54(10), 1422-1427.
17	Robitaille, G., Lapointe, C., Leclerc, D., & Britten, M. (2012). Effect of pepsin-treated bovine and goat caseinomacropeptide on Escherichia coli and Lactobacillus rhamnosus in acidic conditions. Journal of dairy science, 95(1), 1-8.
18	Jacquot, A., Gauthier, S. F., Drouin, R., & Boutin, Y. (2010). Proliferative effects of synthetic peptides from β-lactoglobulin and α-lactalbumin on murine splenocytes. International dairy journal, 20(8), 514-521.
19	Lacroix, I. M., Meng, G., Cheung, I. W., & Li-Chan, E. C. (2016). Do whey protein-derived peptides have dual dipeptidyl-peptidase IV and angiotensin I-converting enzyme inhibitory activities? Journal of functional foods, 21, 87-96.

20	Tavares, T., del Mar Contreras, M., Amorim, M., Pintado, M., Recio, I., & Malcata, F. X. (2011). Novel whey-derived peptides with inhibitory effect against angiotensin-converting enzyme: in vitro effect and stability to gastrointestinal enzymes. Peptides, 32(5), 1013-1019.
21	Tavares, T. G., & Malcata, F. X. (2012). The Portuguese Paradox: Why do some inhabitants of Portugal appear to live so long when their diet is based on whey cheese? Food chemistry, 131(3), 727-729.
22	Lacroix, I. M., & Li-Chan, E. C. (2014). Isolation and characterization of peptides with dipeptidyl peptidase-IV inhibitory activity from pepsin-treated bovine whey proteins. Peptides, 54, 39-48.
